# The Novel Clostridial Neurotoxin Produced by Strain IBCA10-7060 Is Immunologically Equivalent to BoNT/HA

**DOI:** 10.3390/toxins12010009

**Published:** 2019-12-20

**Authors:** Yongfeng Fan, Jason R. Barash, Fraser Conrad, Jianlong Lou, Christina Tam, Luisa W. Cheng, Stephen S. Arnon, James D. Marks

**Affiliations:** 1 Department of Anesthesia and Perioperative Care, University of California San Francisco, Zuckerberg San Francisco General Hospital, 1001 Potrero Ave, San Francisco, CA 94110, USA; frank.fan@ucsf.edu (Y.F.); fraser.conrad@ucsf.edu (F.C.); Jianlong.lou@ucsf.edu (J.L.); 2 Infant Botulism Treatment and Prevention Program, Division of Communicable Disease Control, Center for Infectious Diseases, California Department of Public Health, Richmond, CA 94804, USA; Jason.barash@cdph.ca.gov (J.R.B.); stephen.arnon@cdph.ca.gov (S.S.A.); 3 Foodborne Toxin Detection and Prevention Research Unit, Western Regional Research Center, Agricultural Research Service, United States Department of Agriculture, 800 Buchanan Street, Albany, CA 94710, USA; christina.tam@ars.usda.gov (C.T.); luisa.cheng@ars.usda.gov (L.W.C.)

**Keywords:** monoclonal antibodies, botulinum neurotoxin, botulism, *Clostridium botulinum*, botulinum neurotoxin HA, BoNT/HA

## Abstract

Background: Botulinum neurotoxins (BoNTs) comprise seven agreed-on serotypes, A through G. In 2014, a novel chimeric neurotoxin produced by clostridial strain IBCA10-7060 was reported as BoNT/H, with subsequent names of BoNT/FA or BoNT/HA based on sequence homology of the N-terminus to BoNT/F, the C-terminus to BoNT/A and neutralization studies. The purpose of this study was to define the immunologic identity of the novel BoNT. Methods: monoclonal antibodies (mAbs) to the novel BoNT/H N-terminus were generated by antibody repertoire cloning and yeast display after immunization with BoNT/H LC-H_N_ or BoNT/F LC-H_N_. Results: 21 unique BoNT/H LC-H_N_ mAbs were obtained; 15 from the BoNT/H LC-H_N_ immunized library (K_D_ 0.78 nM to 182 nM) and six from the BoNT/F-immunized libraries (K_D_ 20.5 nM to 1490 nM). A total of 15 of 21 mAbs also bound catalytically inactive BoNT/H holotoxin. The mAbs bound nine non-overlapping epitopes on the BoNT/H LC-H_N_. None of the mAbs showed binding to BoNT serotypes A-G, nor any of the seven subtypes of BoNT/F, except for one mAb that weakly bound BoNT/F5. Conclusions: The results, combined with the chimeric structure and neutralization by anti-A, but not anti-F antitoxin indicate that immunologically the novel BoNT is BoNT/HA. This determination has significant implications for existing countermeasures and potential vulnerabilities.

## 1. Introduction

Botulinum neurotoxins (BoNTs), the most poisonous substances known [1], consist of three functional domains [2]: a binding domain (H_C_), translocation domain (H_N_) and catalytic domain (LC). BoNTs exist as seven accepted serotypes (A–G), defined immunologically by the inability of polyclonal antibody that neutralizes one serotype to neutralize other serotypes [3]. Toxin serotypes A, B, E, and F can be further subdivided into subtypes or genetic variants (A1–A8, F1-9) based on sequence and immunologic differences [4]. Additional toxins with structural homology to BoNT have also been recently identified and include BoNT/X from a Clostridial species and eBoNT/J [5,6] from an Enterococcal species.

In 2014, a novel neurotoxin was reported that was produced by the bivalent *Clostridium botulinum* strain IBCA10-7060, which also produced BoNT/B2 [7,8]. The novel BoNT appeared to be chimeric with a H_C_ most homologous to BoNT/A1 (~84%) and an H_N_ and LC most homologous to BoNT/F5 (~64% and ~81% respectively) [8] (Figure S1). In the initial report, the novel neurotoxin was considered a new serotype, BoNT/H, based on failure of neutralization using the standard mouse bioassay [7] and also using a research antitoxin at antitoxin:toxin ratios as high as 595:1 [7]. Subsequent work demonstrated that the novel BoNT could be neutralized by a combination of anti-A and anti-B research antitoxin at ratios ranging from 20:1 to 200:1 [9,10]. Based on this neutralization and the mosaic structure of the novel toxin with its homology to parts of BoNT/A and BoNT/F5, these authors and others termed the novel toxin BoNT/FA [10,11]. However, the novel BoNT was not neutralized by anti-BoNT/F antitoxin [9,10] and was bound by only one of six anti-BoNT/F monoclonal antibodies (mAbs) and with an affinity more than 8000-fold lower than the affinity for BoNT/F1 (K_D_ = 9.1 pM vs. ~75 nM) [12]. Based on these findings, the novel BoNT has also been termed BoNT/HA [13,14].

We sought to better define the immunologic nature of the novel BoNT by generating a panel of mAbs against the LC-H_N_ portion of the novel toxin and determining their ability to bind other BoNT serotypes. The results indicate that immunologically, the novel BoNT is BoNT/HA, which has significant implications for existing countermeasures and potential vulnerabilities.

## 2. Results

Since the novel BoNT has been termed BoNT/H, BoNT/FA, BoNT/HA and the novel neurotoxin produced by strain IBCA10-7060, for clarity and brevity we will refer to the novel BoNT throughout the results section by the first name used to describe it, BoNT/H [7]. In the discussion and conclusion, we will use the name that is supported by the studies performed, BoNT/HA.

### 2.1. BoNT/H LC-H_N_ Fragment Expression and Mouse Immunization

To focus the immune response on the portion of the novel BoNT not homologous to BoNT/A, the BoNT/H LC-H_N_ gene encoding amino acids 1–859 was cloned into plasmid pET28b, expressed from *Escherichia. coli* and purified by IMAC (Figure S2A). The recombinant BoNT/H LC-H_N_ was bound by the BoNT/H H_N_ mAb 6F5.4 [12] and by anti-His tag IgG by ELISA (Figure S2B). The BoNT/H LC (amino acids 1–444) was produced similarly and was of the expected size by SDS-PAGE (Figure S2A). Mice were immunized with BoNT/H LC-H_N_ and serum harvested at six weeks after the initial immunization. Immune serum bound recombinant BoNT/H LC-H_N_ at dilutions greater than 1:325 as determined by ELISA (Figure S2C).

### 2.2. Isolation and Initial Characterization of mAbs from Mice Immunized with BoNT/H LC-H_N_

To generate antibodies to BoNT/H LC-H_N_, murine V_H_ and V_K_ genes were PCR amplified from cDNA prepared from the splenocytes of immunized mice and cloned into the yeast display vector pYD4 to create a single chain Fv (scFv) antibody gene repertoire as described [12]. The scFv gene repertoire in pYD4 was used to transform *Saccharomyces cerevisiae* EBY100 to create a yeast-displayed scFv library of 5 × 10^7^ (Table 1). scFv display was induced, and the library sequentially sorted for three rounds after staining with BoNT/H LC-H_N_. After the final round of sorting, 96 individual colonies were analyzed for BoNT/H LC-H_N_ binding. The scFv gene of each binding clone was sequenced resulting in 15 unique scFv binding BoNT/H LC-H_N_ (Table 2). Equilibrium dissociation constants (K_D_) of each yeast-displayed scFv for BoNT/H LC-H_N_ were measured by flow cytometry and ranged from 0.78 nM–182 nM with a median K_D_ of 12.5 nM (Table 2).

To confirm that the epitopes bound by BoNT/H LC-H_N_ mAbs exist in the holotoxin, we attempted to measure the K_D_ of yeast displayed scFv for BoNT/H in crude IBCA10-7060 culture supernatants. However, the large amounts of BoNT/B in the supernatants cleaved the scFv from the yeast surface, even in the presence of zinc chelators and protease inhibitors, precluding K_D_ measurement. We therefore purchased commercially available catalytically inactive BoNT/Hi holotoxin. To validate that the recombinant BoNT/Hi was properly folded, we measured the K_D_ of two BoNT/A HC IgG (CR2 and RAZ1) and one BoNT/F mAb (6F5.4) that we have previously shown bound BoNT/H [12]. All three of these mAbs bind conformational epitopes as shown by alanine scanning and/or X-ray crystallography and do not bind linear epitopes [15,16,17]. As shown in Table S1, all three of these mAbs bound BoNT/Hi with a K_D_ indistinguishable from their K_D_ for BoNT/H.

**Figure 1:**
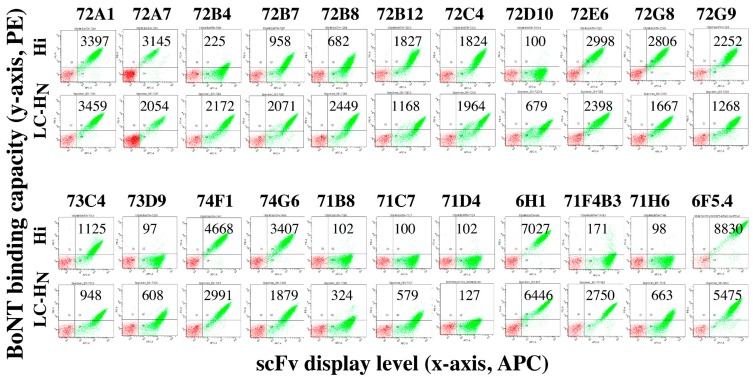
Dot-plots of the binding of single chain Fv (scFv) to botulinum neurotoxins (BoNT)/Hi. The ability of each yeast-displayed scFv to bind BoNT/Hi holotoxin was determined by flow cytometry. Binding of each scFv to BoNT/H LC-H_N_ is shown as a positive control. Binding was detected using mAb 6F5.4 [12] or 6H1 (for 74F1 and 74G6) and is shown on the *y*-axis. The level of scFv display is shown on the *x*-axis. The green gated population are those yeast that display scFv on their surface. Mean fluorescent intensity (MFI) values are shown within each dot-plot. The negative control MFI (yeast stained with secondary antibody only) was 94.2 ± 15.7. A test MFI > two standard deviations from control (>125.6) was considered positive for binding BoNT/H LC-HN or BoNT/Hi.

Thirteen of 15 scFv bound recombinant BoNT/Hi holotoxin, with the two non-binding scFv (72D10 and 73D9) having two of the three lowest affinities for BoNT/H LC-H_N_ (Table 2 and Figure 1). This result indicates that the recombinant BoNT/H LC-H_N_ represents the LC-H_N_ structure as it exists in the holotoxin. Failure of scFvs to bind BoNT/Hi holotoxin could result from buried LC-H_N_ epitopes due to packing of the H_C_ against the H_N_ in the holotoxin, or the scFv might bind epitopes on the BoNT/LC-H_N_ that are not on the holotoxin. The scFv without BoNT/Hi binding also generally had lower affinity, and may not be detected with the current method.

### 2.3. Isolation and Initial Characterization of mAbs from Mice Immunized with BoNT/F LC-H_N_

Since the BoNT/H LC-H_N_ is most homologous to the BoNT/F5 subtype, we sorted five yeast-displayed scFv libraries generated from mice immunized with recombinant BoNT/F domains (Table 1) [18] to determine if rare cross-reactive BoNT/H LC-H_N_ mAbs could be isolated. After sorting on BoNT/H LC-H_N_, a total of six unique scFv were isolated with K_D_ values of 20.5 nM–1490 nM (median of 127 nM, Table 2). Only one of six scFv bound recombinant BoNT/Hi holotoxin (Figure 1). The five non-binding scFv also generally had lower affinity for BoNT/H LC-H_N_ than scFv that bound BoNT/Hi from the BoNT/H LC-H_N_ library. One of the non-binding scFv had measurable binding to BoNT/Hi after its affinity was increased four-fold to 34.6 nM (Table 2).

### 2.4. The BoNT/H LC-H_N_ scFv mAbs Bind Nine Non-Overlapping Epitopes

**Figure 2:**
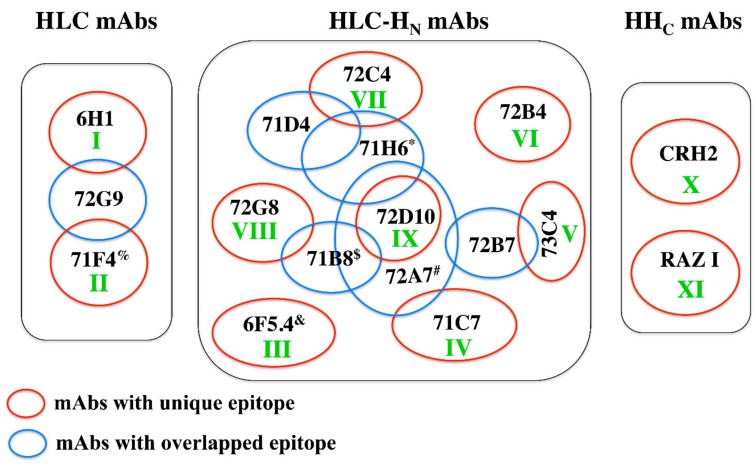
Map of BoNT/H scFv domain specificity and epitope overlap. scFv were mapped to the BoNT/H LC or LC-H_N_ based on their ability to bind each recombinant domain as determined by flow cytometry. Since recombinant H_N_ was not produced, LC-H_N_ binding scFv cannot be distinguished from H_N_ binding scFv, except for scFv 74F1 and 74G6 which have epitopes that overlap with the H_N_ binding scFv 6F5.4. The mAb clusters were based on ability of each scFv to bind simultaneously to BoNT/H LC, LC-H_N_ or BoNT/A H_C_ as determined by flow cytometry. The BoNT/H LC-H_N_ scFv bound nine non-overlapping epitopes (I–IX), shown as red circles defined by a specific scFv. CRH2 and RAZ1 bind two unique epitopes on the BoNT/H H_C_. * shared epitope with scFv 73D9; # shared epitope with scFv 72E6 and scFv 72B12: $ shared epitope with scFv 72B8; & shared epitope with scFv 74F1 and scFv 74G6; % shared epitope with scFv 72A1.

The 21 BoNT/H LC-H_N_ scFv from the BoNT/H LC-H_N_ and BoNT/F LC-H_N_ immunized mice were clustered into non-overlapping epitope groups based on the ability of soluble scFv to bind BoNT/H LC-H_N_ after capture by yeast-displayed scFv (Figure 2 and Table 2). The 15 scFv from the BoNT/H LC-H_N_ library clustered into eight non-overlapping epitopes groups (groups II-IX). Two scFv (72A1 and 72G9) bound an overlapping epitope on the BoNT/H LC (Table 2 and Figure S3) while the remaining 13 bound the recombinant LC-H_N_ but not the LC. The epitopes of scFv 74F1 and 74G6 were inferred to be on the BoNT/H H_N_ since their epitopes overlapped with H_N_ specific mAb 6F5.4 [12]. The remaining 11 scFv either bound epitopes spanning the LC-H_N_ or were H_N_ specific. Since we were unable to produce recombinant BoNT/H H_N_, we cannot distinguish between these two possibilities. Two of the six scFv (6H1 and 71F4) from the BoNT/F LC-H_N_ libraries bound the BoNT/H LC (Table 2 and Figure S3). Overall, the epitopes of five of the scFv overlapped with one or more scFv from the BoNT/H LC-H_N_ libraries, while one of the scFv (6H1) defined a new epitope (I) (Figure 2).

### 2.5. mAbs Binding BoNT/H LC-H_N_ do not Bind BoNT Serotypes A–G

**Figure 3:**
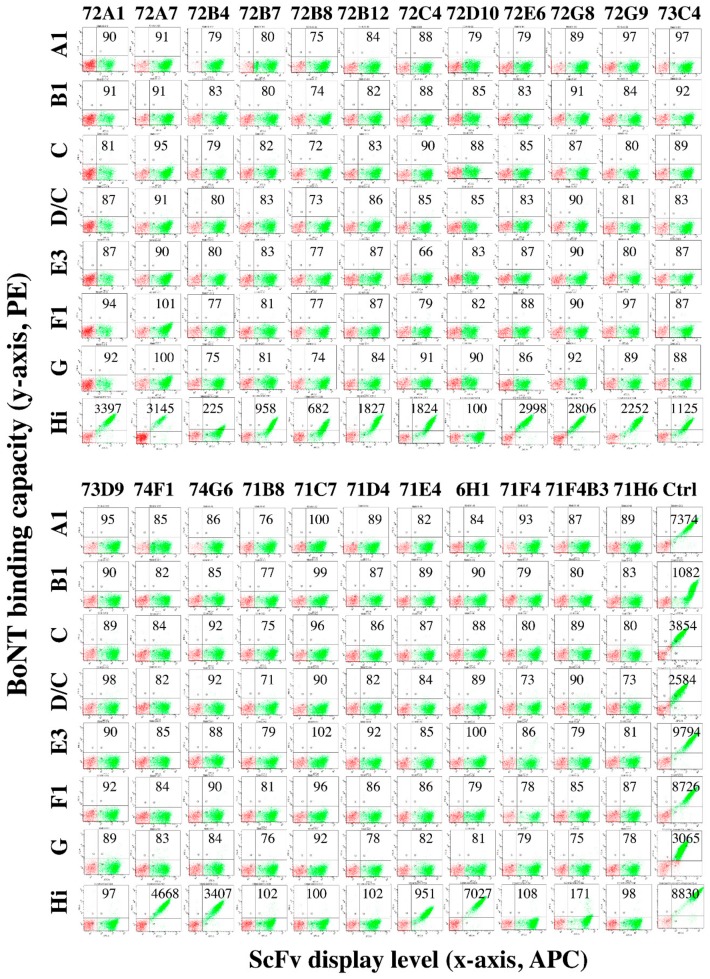
Dot-plots of the specificity of BoNT/H LC-H_N_ scFv for BoNT serotypes A, B, C, D/C, E, F, G and Hi. Yeast-displayed BoNT/H LC-H_N_ scFv were incubated with 50 nM of the indicated BoNT holotoxin. BoNT binding was detected with IgG mAbs RAZ1/CR2 for BoNT/A1, 1B10.1/B6.1 for BoNT/B1, 4C2/4C4.2 for BoNT/C and D/C, 3E2/4E17.1 for BoNT/E3, hu6F11/hu6F13.4 for BoNT/F1, 7G1.1/7G3.1 for BoNT/G and 6F5.4 and 6H1 for BoNT/Hi followed by PE-conjugated goat-anti human IgG. Yeast-displayed 6F5.4 was used for the positive control for BoNT/A1, B1, E3, F1, Hi and yeast-displayed 4C2 or 7G1.1 was used for BoNT/C, D/C and BoNT/G. None of the scFv bound any BoNTs except BoNT/H. The level of BoNT binding is indicated on the Y-axis and the level of scFv display is shown on the X-axis. The gated populations shown in green are those yeasts displaying scFv on their surface. Mean fluorescent intensity (MFI) values are shown within each dot-plot. The negative control MFI (yeast stained with secondary antibody only) was 94.2 ± 15.7. A test MFI > two standard deviations from control (>125.6) was considered positive for binding BoNT.

To determine the specificity of the 21 BoNT/H LC-H_N_ scFv, each yeast-displayed scFv was stained with 100 nM of BoNT/A1, B1, C, D/C, E3, F1 and G holotoxin and binding detected by flow cytometry. None of the 21 BoNT/H LC-H_N_ scFv bound any other BoNT serotypes, including BoNT/F1 (Figure 3), indicating that BoNT/H LC-H_N_ is immunologically distinct from the seven other serotypes. This absence of binding to BoNT/F for scFv from mice immunized with BoNT/F LC-H_N_ indicates that the scFv selected on BoNT/H LC-H_N_ do not represent rare scFv binding both BoNT/H and BoNT/F but rather unique BoNT/H specificities arising most likely from non-cognate immunoglobulin VH-VL pairings created during library construction.

**Figure 4:**
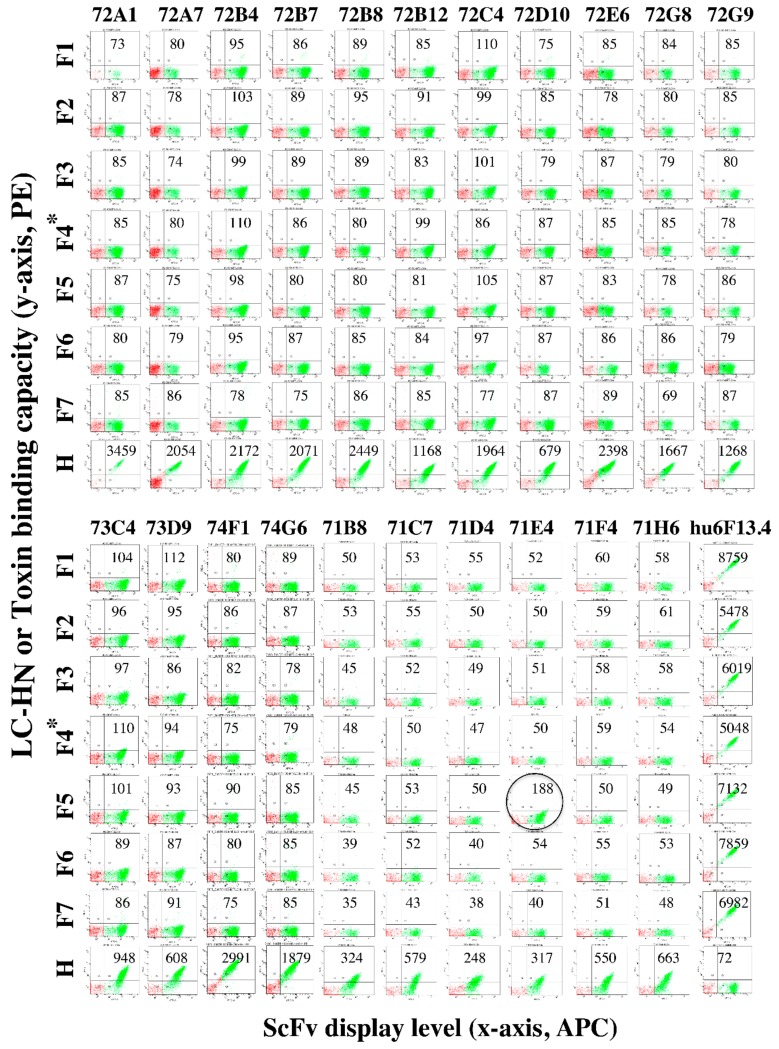
Dot plots of the specificity of BoNT/H LC-H_N_ scFv for BoNT/F subtypes. Yeast-displayed BoNT/H LC-H_N_ scFv were incubated with 100 nM of the indicated BoNT/F LC-H_N_ subtype, BoNT/F4 holotoxin (indicated with an * in the Figure) or BoNT/H LC-H_N_. Yeast-displayed hu6F13.4 was used for a control. BoNT/F subtype binding and BoNT/H LC-H_N_ binding was detected with IgG 6F5.4 and 6H1 (for 74F1 and 74G6). Only scFv 71E4 (result circled) showed weak binding to BoNT/F5 LC-H_N_, while other scFv did not show any binding to BoNT/F subtypes. The level of BoNT binding is indicated on the *y*-axis and the level of scFv display is shown on the *x*-axis. The gated populations shown in green are those yeasts displaying scFv on their surface. Mean fluorescent intensity (MFI) values are shown within each dot-plot. The negative control MFI (yeast stained with secondary antibody only) was 94.2 ± 15.7. A test MFI > two standard deviations from control (>125.6) was considered positive for binding BoNT.

Since the BoNT/H LC-H_N_ is most homologous with the BoNT/F serotype, we tested the ability of each mAb to bind each of seven BoNT/F subtypes. None of the mAbs derived from BoNT/H LC-H_N_ immunization bound any BoNT/F subtype, including the light chain binders 72A1 and 72G9 (Figure 4). One of the six mAbs derived from BoNT/F LC-H_N_ immunization (71E4) weakly bound BoNT/F5LC-H_N_ (Figure 3) with a K_D_ 5.6-fold lower than the K_D_ for BoNT/H LC-H_N_ (Table 2). When the affinity of 71E4 for BoNT/H LC-H_N_ was increased 24.5-fold to obtain scFv 6H1, the affinity for BoNT/F5 LC-H_N_ also increased 30.2-fold. However, neither the 71E4 scFv nor the higher affinity 6H1 scFv recognized other BoNT/F subtypes (Figure S4). The cross-reactivity of 71E4 and 6H1 for BoNT/H and only BoNT/F5 LC suggests that there exist common epitopes on the light chain of BoNT/H and BoNT/F5 that are not found on the other BoNT/F subtypes or other BoNT serotypes, consistent with an amino acid identity of 80.1% for BoNT/H LC and BoNT/F5 LC, and less than 50% between BoNT/H LC and other BoNT/F subtypes. However, the immunological similarity of BoNT/H and BoNT/F5 LC is relatively limited. scFv 72A1 and 72G9 also bound BoNT/H LC with high affinity (K_D_ 3.08 nM and 6.62 nM, Table 2), but did not bind BoNT/F5LC-H_N_ (Figure 4). Similarly, the scFv 71F4 binds BoNT/H LC (K_D_ on yeast 128 nM) but not BoNT/F5LC-H_N_ even after its affinity was improved to 34 nM (71F4B3) (Figure S4).

BoNT/H H_N_ shares only 61.7% and 64.1% amino acid sequence identity with BoNT/F1 H_N_ and BoNT/F5 H_N_ respectively [11]; thus the immunological differences are likely greater than for the BoNT/F5 LC. While mAb 6F5.4 bound the H_N_ domain of BoNT/H and all BoNT/F subtypes with high affinity [12], neither of the BoNT/H H_N_ scFv that had overlapping epitopes with 6F5.4 (74F1 and 74G6) bound any BoNT/F subtype (Figure 4 and Figure S5). To determine whether BoNT/H and BoNT/F5 shared additional epitopes, the BoNT/H immune yeast-displayed library was sorted after incubation with BoNT/F5 LC-H_N_. Two scFv were identified, 77H2 and 78A2, that bound BoNT/F5 LC-H_N_ with affinities of 80.9 nM and 110.4 nM respectively. However, neither of these scFv bound BoNT/H LC-H_N_ (Figure S6).

### 2.6. Some mAbs can Bind both BoNT/A H_C_ and BoNT/H

The BoNT H_C_ comprises two functional domains H_CN_ and H_CC_. The BoNT/A H_CC_ and BoNT/H H_CC_ have greater sequence identity (92.2%) than the BoNT/A H_CN_ and BoNT/H H_CN_ (73.1%) [7,12]. We previously evaluated four BoNT/A H_C_ mAbs each binding non-overlapping epitopes (RAZ1, S25, CR2, and B4) for binding to BoNT/H. The two H_CC_ binding mAbs (RAZ1 and S25) bound BoNT/H with affinities comparable to those for BoNT/A binding. One of the mAbs binding the BoNT/A H_CN_ (B4) did not bind BoNT/H and the other, CR2, bound BoNT/H with a 400-fold lower affinity than BoNT/A [12]. To understand differences in the H_CN_ epitope of CR2, we selected four yeast-displayed libraries of mutants of the CR2 parental antibody CR1 for higher affinity binding to BoNT/H, followed by additional rounds of random mutagenesis to further increase affinity. The resulting antibody, CRH2, had a 19-fold increased affinity for BoNT/H (K_D _ 5.37 × 10^−9^ M vs. 2.9 × 10^−10^ M) but a 100-fold reduction in K_D_ for BoNT/A1 (from 8.8 × 10^−9^ M to 8.9 × 10^−7^) demonstrating the challenges generating cross reactive antibodies to this BoNT epitope.

## 3. Discussion

A panel of 21 scFv mAbs binding the novel BoNT produced by *C. botulinum* strain IBCA10-7060 were isolated which bound nine non-overlapping epitopes spanning the H_N_ and the LC domains. Despite this broad epitopic coverage, none of the 21 mAbs bound the other 7 BoNT serotypes except for one weakly binding the LC of BoNT/F5. We employed two selection strategies to enrich for potentially rare mAbs that bound both the novel BoNT and BoNT/F, the serotype whose subtype F5 has the closest LC-H_N_ homology. In the first of these strategies, we used BoNT/F immune libraries and selected for BoNT/H LC-H_N_ binding. Six mAbs binding the novel BoNT were obtained, but none bound any of the seven BoNT/F subtypes except the one mAb mentioned above that weakly bound to BoNT/F5 LC. In the second approach, the BoNT/H LC-H_N_ immune library was selected for binding to the BoNT/F5 LC-H_N_. Two mAbs were obtained that bound BoNT/F5 LC-H_N,_ but neither bound BoNT/H LC-H_N_. In both instances, the mAbs likely resulted from light chain shuffling which occurs during library construction and which creates new specificities that are not present in the original immune repertoire.

The results strongly indicate that the LC-H_N_ of the novel BoNT is immunologically distinct from the other BoNT serotypes and thus should be termed BoNT/H LC-H_N_. This conclusion is supported by several other studies. First, only one of six mAbs (6F5.1) binding BoNT/F LC or H_N_ domains bound the novel BoNT, and its novel toxin K_D_ was 800-fold lower than the BoNT/F K_D_. This mAb, 6F5.1, binds an epitope at the tip of the H_N_ that is shared by BoNT/A, B, E, F and the novel BoNT, and is the only mAb we have identified out of more than 100 mAbs studied that has this multi-serotype specificity [17]. Second, multiple studies have shown that BoNT/F polyclonal antisera alone does not neutralize the novel BoNT in mice [7,9]. This conclusion is consistent with the limited amino acid identity between the BoNT/H LC and H_N_ and BoNT/F1 LC and H_N_ (48.8% and 61.7% respectively, Figure S1), differences that are no different than the amino acid percentage identities between the seven BoNT serotypes (32.6–64.1%) [8]. Our use of a large panel of mAbs binding both the LC and H_N_ provides confidence that both of these domains are immunologically distinct from the other BoNT serotypes. Such conclusions are less strongly supported using polyclonal antisera since there could be considerable epitopic bias from immunodominant epitopes. The H_CC_ and H_CN_ domains of the novel BoNT were bound by two of two and one of two anti-BoNT/A mAbs, respectively, a finding consistent with the amino acid percentage identities of each of these domains to BoNT/A1 (92.2% and 73.1% respectively) [12]. The novel BoNT is also neutralized in mice by anti-BoNT/A research polyclonal antitoxins, although 16-fold to 1080-fold less potently than BoNT/A1 [7,9]. Based on the above findings, immunologically the novel BoNT is BoNT/HA.

There is both precedent and value for the use of such chimeric nomenclature. For example, in 1924 it was found that anti-sera produced from “BoNT/C” from an avian species could neutralize BoNT/C from other avian strains and neutralize the Seddon type C strain causing botulism in cattle in Australia; however anti-sera from the Seddon strain did not neutralize avian BoNT/C [19]. This neutralization discrepancy was explained when DNA sequencing revealed the existence of chimeras between BoNT/C and BoNT/D that have been termed BoNT CD and BoNT DC. In BoNT CD, the N-terminal 2/3 is type C in sequence and the C-terminal 1/3 type D in sequence [20]. In BoNT DC the N-terminal 2/3 is type D in sequence and the C-terminal 1/3 is type C in sequence [21]. While BoNT CD and BoNT DC are not considered new serotypes, the use of the chimeric nomenclature both provides structural insights as well as helps explain otherwise inexplicable neutralization results. Such is the case with BoNT/HA, the nomenclature tells us that the N-terminus is unique and that neutralization by BoNT/A anti-sera will be less potent than anticipated (see ref [22] and conclusions below). The use of the chimeric nomenclature does beg the question how much of the structure needs to be immunologically unique (or different) before it is defined as a novel chimera. We would propose using some combination of precedent, the BoNT CD and DC studies where there is a domain level difference in structure, and whether there is an impact on potency of neutralization compared to the reference serotype. BoNT/HA meets both criteria.

The BoNT/HA gene is located at a unique site in the strain IBCA10-7060 chromosome that resulted from an insertion event facilitated by flanking IS 110 elements [8]. The chimeric nature of BoNT/HA with its sequence divergence from all known BoNTs except in the H_CC_ region suggests that BoNT/HA is derived from an ancestral BoNT that has undergone multiple rounds of recombination and sequence mutation [8,23]. Of note, the higher homology of the BoNT/F5 LC to the BoNT/HA LC (80%) compared to the other BoNT/F subtypes (46.3–48.3%) suggests that BoNT/F5 may also represent one of these ancestral recombination events and would more accurately be termed BoNT/HF. This interpretation is consistent with the unique substrate cleavage site common to the BoNT/HA and BoNT/F5 LC that no other BoNTs have [24]. In addition, a Simplot showing that the first 1500 nucleotides encoding the BoNT/F5 LC gene have low similarity to the same nucleotides in the other six BoNT/F subtypes led to the conclusion that BoNT/F5 was in fact an unrecognized mosaic (“hybrid”) of the H_C_-H_N_ of BoNT/F2 and an/H-like LC [8].

## 4. Conclusions

The above findings have implications for the use of existing BoNT countermeasures and the development of new countermeasures. 500-fold more equine botulinum antitoxin (BAT, serotypes A-G) [22] was required to neutralize BoNT/HA than to neutralize BoNT/A1 [9]. This is expected given that only 1/6 of BoNT/HA has high identity with any of the seven serotypes A-G used to generate BAT (the H_CC_ of BoNT/HA with BoNT/A). Based on relative potencies, a one-vial dose of BAT in humans has been calculated to neutralize 50–54 mouse LD_50_s of BoNT/HA per mL of human serum [9,12]. Serum concentrations of BoNT in patients with foodborne botulism in one study were reported as high as 32 mouse LD_50_s/mL [25], with the highest foodborne botulism serum concentration reported being 1800 mouse LD_50_/mL of BoNT/A [26]. Serum concentrations of BoNT expected from bioterrorism are unknown. Comparing the estimated BoNT/HA neutralizing activity of BAT (<54 MLD_50_s/mL) with reported BoNT serum concentrations (up to 1800 LD_50_s/mL) indicates that for some patients a single vial of BAT might be inadequate. In addition, the ability of BoNT to persist in serum for as long as 25 days after exposure [26,27] together with the short serum half-life (8.6 h) of the anti-BoNT/A component of BAT [22] heightens the need to anticipate relapse and to consider early BAT re-administration where possible [28].

As a possible alternative to BAT, NTM-1631 [29], a combination of two human and one humanized BoNT/A mAbs (formerly XOMA 3AB), has been calculated to be at least ten times more potent than BAT for BoNT/HA using the mouse neutralization assay [12]. NTM-1631 has a serum half-life of >10 days and was well tolerated with no significant adverse events in a Phase 1 clinical trial [29]. Hence NTM-1631 could be developed as a treatment for botulism due to BoNT/HA.

Finally, if the ancestral BoNT that evolved into BoNT/HA still exists in nature and can cause botulism, it is likely that existing antitoxins would not neutralize it. The mAbs described here could be used for detection of the ancestral BoNT and could be developed into therapeutic antitoxins against both the ancestral BoNT and natural BoNT/HA or bioengineered derivatives.

## 5. Materials and Methods

### 5.1. Materials

Full length, genetically inactive BoNT/H with E227Q and Y366F mutation (BoNT/Hi) was purchased from Toxogen GmbH (Hannover, Germany). The pure toxins BoNT/A1, B1, C, D/C, E3, F1, and G were purchased from Metabiologics Inc. (Madison, WI, USA). mAbs 6H1 and 6F5.4 [12] were expressed from CHO cells as described [30]. All secondary antibodies were purchased from Jackson ImmunoResearch Laboratories (West Grove, PA, USA).

### 5.2. Protein Expression and Purification

The BoNT/H fragments BoNT/H LC (1–444) and HLC-H_N_ (1–859) and soluble scFv were expressed and purified as described [18]. For ELISA, 100 μL of serially diluted BoNT/H LC-H_N_ was coated on a plate, with F1 LC-H_N_ as a control, followed by incubation with mAb 6F5.4 or anti-His tag IgG (1 μg/mL) with detection by using HRP-labeled goat anti-human or mouse IgG (0.1 μg/mL, Jackson ImmunoResearch) and 2,2’-azino-bis(3-ethylbenzothiazoline-6-sulphonic acid) (ABTS) as substrate.

### 5.3. Ethics Statement

The USDA Institutional Animal Care and Use Committee approved the animal care and use protocol to conduct the animal studies reported here under an IACUC-approved protocol (16-1) with an approval date of 7/27/2016 in compliance with the Animal Welfare Act, PHS Policy.

### 5.4. Mouse Immunization, Spleen Harvest, and Antiserum Titer

Mice were immunized with BoNT/H LC-H_N_ three times. Briefly, 0.25–2.5 μg/mouse of BoNT/H LC-H_N_ was intraperitoneally injected with adjuvant (Sigma, Burlington, MA, USA) at day one with boosting on days 21 and 42. Blood was drawn at the submandibular site from mice ten days after their final immunization and analyzed for binding to BoNT/H LC-H_N_ by ELISA. Spleens from mice that had positive ELISA results were used to extract mRNA for scFv library construction.

### 5.5. scFv library Construction and FACS Sorting

The BoNT/H LC-H_N_ scFv yeast display library was constructed as described [31]. BoNT/H specific binders were isolated using three rounds of FACS sorting in which 100 nM of BoNT/H LC-H_N_ was used for yeast incubation, followed by Alexa-647-conjugated 6F5.4 IgG (6F5.4-647) for labeling. In parallel, yeast cells were labeled with BoNT/H LC-H_N_ and co-stained with 6H1-647 and 6F5.4-488, from which 6F5.4 overlapping mAbs were isolated (6F5.4 negative but 6H1 positive colonies). In separate experiments, the BoNT/H LC-H_N_ immunized library was also stained with 100 nM of BoNT/F5 LC-H_N_ followed by incubation with mAb 6F5.4-647 to isolate BoNT/F5 specific mAbs. Individual colonies were picked from SD-CAA plates, cultured and scFv display induced for further characterization [31]. Libraries made from mice immunized with BoNT/F fragments [18] were sorted as described above (Table 1).

### 5.6. scFv binding Confirmation and Affinity Determination

Binding of individual yeast-displayed scFv was confirmed using BoNT/H LC-H_N_ and full-length BoNT/Hi by using flow cytometry. Flow cytometry was used because it allows measurement of the scFv dissociation equilibrium constant (K_D_) without the need to subclone, express and purify the scFv as would be required for ELISA or surface plasmon resonance. The dissociation equilibrium constant (K_D_) of scFv was determined by using flow cytometer as described [18].

### 5.7. Epitope Overlap Determination

Epitope groups on BoNT/H surface were deduced from competition assays as described [18]. Briefly, yeast-displayed scFvs were incubated with 100 nM of BoNT/H LC-H_N_ followed by incubation with soluble myc-tagged scFv. Anti-myc antibody was added for detection of soluble scFv binding and the MFI measured by flow cytometry. A positive signal indicated that both mAbs bound simultaneously.

### 5.8. MAb Affinity Maturation

Spiked oligonucleotide mutagenesis, light chain shuffling and error prone PCR-mediated random mutagenesis were used for affinity maturation of scFv 71E4, 71F4, and CR1. (See Supplementary Materials).

### 5.9. Measurement of Solution Phase Affinity at Equilibrium

For mAbs CR2, RAZ1 and 6F5.4, the solution phase affinity at equilibrium and binding kinetics were measured using flow fluorimetry in a KinExA (Sapidyne, Boise, ID, USA) as previously described [10,25] except that BoNT/Hi toxin was used. BoNT/Hi toxin solution was studied at a concentration estimated to be >10-fold above the value of the equilibrium dissociation binding constant [K_D_] of the mAb for the toxin to generate a concentration-controlled curve for greater accuracy in measuring BoNT/Hi concentrations. mAb-containing solutions were serially diluted in a constant concentration of culture filtrate, from >10-fold above to <0.1-fold below the estimated BoNT/Hi concentrations, to capture a complete binding curve. After equilibrium was achieved, samples were passed over a flow cell packed with Sepharose 4 Fast Flow beads (GE Healthcare, Marlborough, MA, USA) covalently coupled with the measuring mAb. An Alexa-647-labeled mAb binding a nonoverlapping BoNT epitope was then passed over the flow cell, producing a signal proportional to the free BoNT in each sample. An analysis curve yielding values for K_D_ and the binding concentrations of the BoNTs was generated using KinExA Pro software (version 4.2.14, Sapidyne, Boise, ID, USA) and a 1:1 reverse-binding model.

## 6. Patents

The antibodies described here are the subject of patent application US20180208680A1, “Antibodies for Botulinum Neurotoxins” inventors James D. Marks, Yongfeng Fan, Jianlong Lou, Maria Consuelo Garcia Rodriguez, Shude Yan, Isin Nergiz Geren, Wenwu Zhai, Subhendu Chakraborti.

## Figures and Tables

**Table 1:** Yeast-displayed scFv libraries used for monoclonal antibodies (mAb) isolation. The BoNT/F libraries are described in Reference [18].

Library Name	Immunogens	Library Size
BoNT/F libraries	BoNT/F1H_C_ and F1 holotoxin BoNT/F3 LC-H_N_ BoNT/F5 LC-H_N_ BoNT/F6 LC-H_N_ BoNT/F7 LC-H_N_	2 × 10^8^
BoNT/H library	BoNT/H LC-H_N_	5 × 10^7^

**Table 2:** The characteristics of selected BoNT/H LC-H_N_ mAbs.

	Domain Specificity	BoNT/F Binding^1^	K_D_ on Yeast (nM)		Epitope Group
			H LC-H_N_	BoNT/Hi	
**MAbs from BoNT/H Library**					
72A1	H LC	No	3.08	2.41	II
72A7	H LC-H_N_	No	0.78	1.79	IX
72B4	H LC-H_N_	No	12.5	>200	VI
72B7	H LC-H_N_	No	25.1	32.85	V/IX
72B8	H LC-H_N_	No	14.2	52.4	IX
72B12	H LC-H_N_	No	29.4	19.5	IX/IV
72C4	H LC-H_N_	No	17.1	43.5	VII
72D10	H LC-H_N_	No	40.8	No Binding	IX
72E6	H LC-H_N_	No	0.99	0.88	IX
72G8	H LC-H_N_	No	5.00	2.4	VIII
72G9	H LC	No	6.62	53.9	I/II
73C4	H LC-H_N_	No	182	>100	V
73D9	H LC-H_N_	No	30.0	No Binding	VII/IX
74F1	H H_N_	No	1.77	2.18	II
74G6	H H_N_	No	4.05	5.50	III
**MAbs from BoNT/F Libraries**					
71B8	H LC-H_N_	No	1490	No Binding	VIII/IX
71C7	H LC-H_N_	No	38.8	No Binding	IV
71D4	H LC-H_N_	No	184.8	No Binding	III
71E4	H LC	F5 LC-H_N_	70.65 (398^4^)	68.8	I
6H1^2^			2.89 (13.2^4^)	3.07	
71F4	H LC	No	124.4	No Binding	II
71F4B^3^			34.6	>200	
71H6	H LC-H_N_	No	20.5	No Binding	VII/IX

^1^ No binding of BoNT/Hi means the scFv did not show detectable binding when tested with 200nM BoNT/Hi. ^2^ mAb 6H1 is an affinity improved derivative of mAb 71E4; ^3^ mAb 71F4B3 is an affinity improved derivative of mAb 71F4. ^4^ the K_D_ values in parentheses are for the K_D_ for binding to BoNT/F5 LC-H_N_.

## Supplementary Materials

The following are available online at https://www.mdpi.com/2072-6651/12/1/9/s1, Table S1: mAbs 6F5.4, CR2 and RAZ1 bind BoNT/H and BoNT/Hi with comparable dissociation equilibrium constants (K_D_). Figure S1: amino acid identity of botulinum neurotoxin type H (BoNT/H) holotoxin and domains compared to BoNT/A1, BoNT/F1 and BoNT/F5. Figure S2: analysis of BoNT/H fragments and results of mouse immunization. Figure S3: dot plots of the specificity of BoNT/H LC binding scFv. Figure S4: affinity maturation of 71E4 and 71F4 did not extend their binding profile. Figure S5: dot plots of the binding of BoNT/H H_N_ scFv to BoNT/F subtypes and BoNT/A. **Figure S6**: BoNT/F5 LC-H_N_ scFv 77H2 and 78A5 bind the H_N_ domain and do not bind BoNT/H LC-H_N_.
