# Supplementary material for: The Novel Clostridial Neurotoxin Produced by Strain IBCA10-7060 Is Immunologically Equivalent to BoNT/HA
Source: Toxins (Basel). 2019 Dec 20;12(1):9. doi: 10.3390/toxins12010009 (PMC7020490; doi:10.3390/toxins12010009)
Supplement: Supplementary file 1 [file toxins-12-00009-s001.pdf]

Supplementary Materials: The Novel Clostridial Neurotoxin Produced by Strain IBCA10-7060 Is Immunologically Equivalent to BoNT/HA

Yongfeng Fan, Jason R. Barash, Fraser Conrad, Jianlong Lou, Christina Tam, Luisa W. Cheng, Stephen S. Arnon and James D. Marks

Table S1. mAbs 6F5.4, CR2 and RAZ1 bind BoNT/H and BoNT/Hi with comparable dissociation equilibrium constants (K_D). Confidence intervals are shown in parentheses. Values for BoNT/H are from Fan et al. [1].

Antibody	BoNT Domain Bound	IgG K_D by KinExA ($\times 10^{-9}$ M)	
		BoNT/H	BoNT/Hi
6F5.4	H_N	0.148 (0.172–0.121)	0.097 (0.121–0.074)
CR2	H_{CN}	5.37 (4.05–6.80)	7.17 (5.78–8.80)
RAZ1	H_{CC}	0.005 (0.003–0.007)	0.006 (0.005–0.007)

BoNT/ Holotoxin

	BoNT/A1	BoNT/F1	BoNT/F5
BoNT/H	49.8%	52.8%	61.8%
BoNT/A1		39.3%	38.5%
BoNT/F1			69.9%

BoNT/LC

	A1LC	F1LC	F5LC
HLC	34.2%	48.8%	80.1%
A1LC		33.8%	32.0%
F1LC			47.3%

BoNT/HC

	A1HC	F1HC	F5HC
HHC	61.1%	55.3%	55.4%
A1HC		44.5%	44.0%
F1HC			82.3%

BoNT/H_N

	$A1H_N$	$F1H_N$	$F5H_N$
HH_N	40.3%	61.7%	64.1%
$A1H_N$		39.2%	41.0%
$F1H_N$			81.0%

BoNT/H_C

	$A1H_C$	$F1H_C$	$F5H_C$
HH_C	81.4%	49.4%	47.3%
$A1H_C$		49.9%	47.4%
$F1H_C$			83.5%

BoNT/H_{CN}

	$A1H_{CN}$	$F1H_{CN}$	$F5H_{CN}$
HH_{CN}	73.1%	55.4%	52.7%
$A1H_{CN}$		56.7%	52.6%
$F1H_{CN}$			81.9%

BoNT/H_{CC}

	$A1H_{CC}$	$F1H_{CC}$	$F5H_{CC}$
HH_{CC}	92.2%	41.1%	40.2%
$A1H_{CC}$		40.8%	40.6%
$F1H_{CC}$			85.4%

Figure S1. Amino acid identity of botulinum neurotoxin type H (BoNT/H) holotoxin and domains compared to BoNT/A1, BoNT/F1 and BoNT/F5. The table indicates the amino acid percentage identity between the sequence of BoNT/H holotoxin and its domains and the other BoNTs indicated (adapted from Fan et al. [1]).

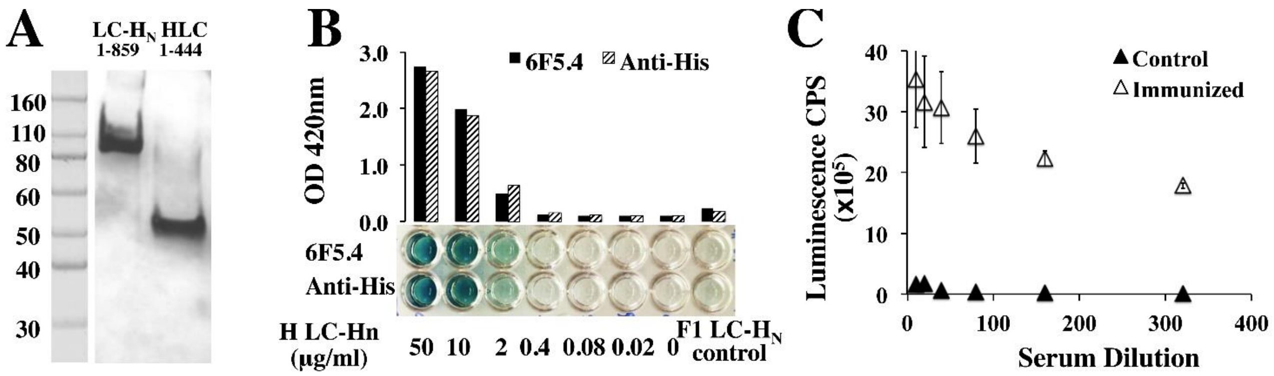

Figure S2. Analysis of BoNT/H fragments and results of mouse immunization. (**A**) SDS-PAGE analysis of the BoNT/H LC (1–444) and BoNT/H LC-HN (1–859). Domains were expressed from BL-21 cells and purified by immobilized metal affinity chromatography. (**B**) Immunoreactivity of hexahistidine tagged BoNT/H LC-HN. Microtitre plate wells were coated with the indicated amount of recombinant BoNT/H LC-HN and binding of anti-His mAb or BoNT/H LC-HN specific mAb 6F5.4 Fan et al. [1] was determined by ELISA. Recombinant F1 LC-HN was used as a negative control. (**C**) **I**mmune response of mice immunized with recombinant BoNT/H LC-HN. Serial dilutions of post-immunization and control mice was analyzed for binding to recombinant BoNT/H LC-HN by ELISA.

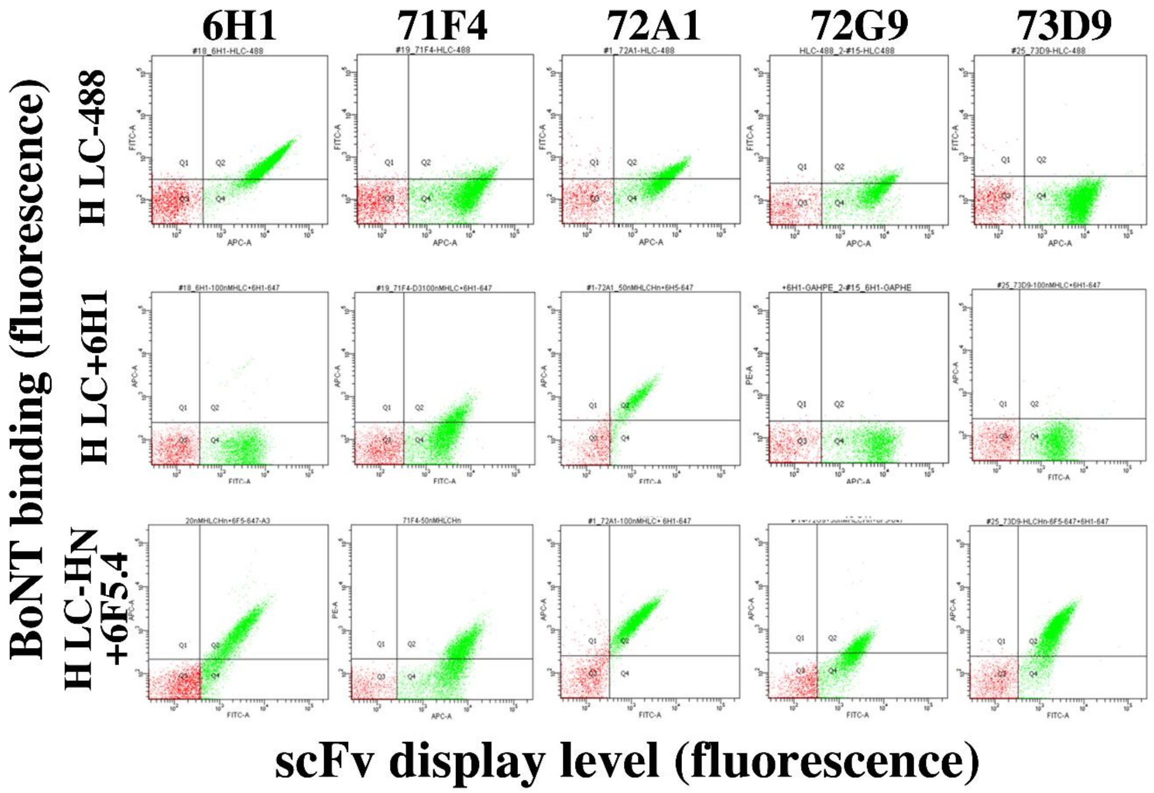

Figure S3. Dot plots of the specificity of BoNT/H LC binding scFv. Upper **panel:** Identification of LC specific BoNT/H scFv. Yeast-displayed scFv were incubated with 100 nM recombinant BoNT/H LC labelled with AlexaFluor-488 and binding detected by flow cytometry. scFv 73D9 was included as a scFv not binding BoNT/H LC. Middle panel: Overlap of LC specific scFv epitopes with the epitope of scFv 6H1. The indicated yeast-displayed scFv in each column was incubated with 100 nM unlabeled BoNT/H LC and the ability of Alexa-647 conjugated IgG 6H1 to bind was determined by flow cytometry. The epitope of scFv 72G9 overlaps with the epitope of 6H1 while the epitope of 72A1 and 71F4 does not. Yeast-displayed 6H1 scFv serves as a control for the assay, which was not detected

with the IgG 6H1. Lower panel: All the scFv, including 73D9, bind BoNT/H LC-HN when detected with AlexaFluor-647 conjugated 6F5.4.

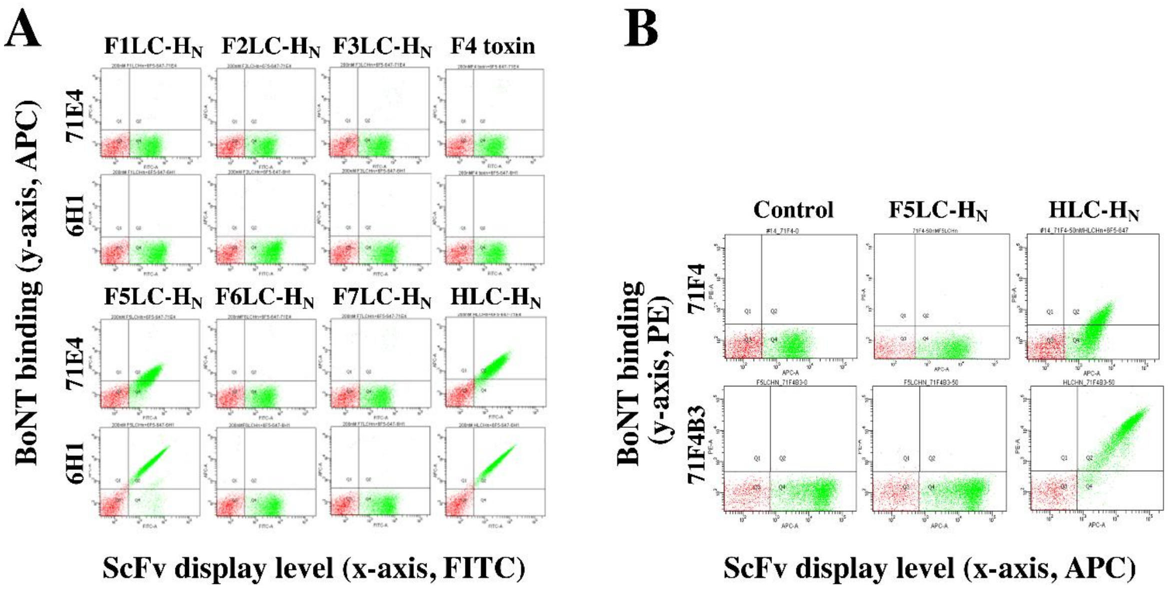

Figure S4. Affinity maturation of 71E4 and 71F4 did not extend their binding profile. (**A**) scFv 6H1 from a 71E4 chain-shuffling library had stronger staining due to higher affinity for both BoNT/H LC-HN (70.65 nM vs. 2.89 nM) and BoNT/F5 LC-HN (398 nM vs. 13.2 nM) but still did not bind the other BoNT/F subtypes. (**B)** Higher affinity scFv 71F4B3 still does not bind BoNT/F5LC-HN. scFv 71F4B3 from a 71F4 chain-shuffling library had stronger staining due to increased affinity for BoNT/H LC-HN (124 nM vs. 34 nM) but still did not bind BoNT/F5LC-HN.

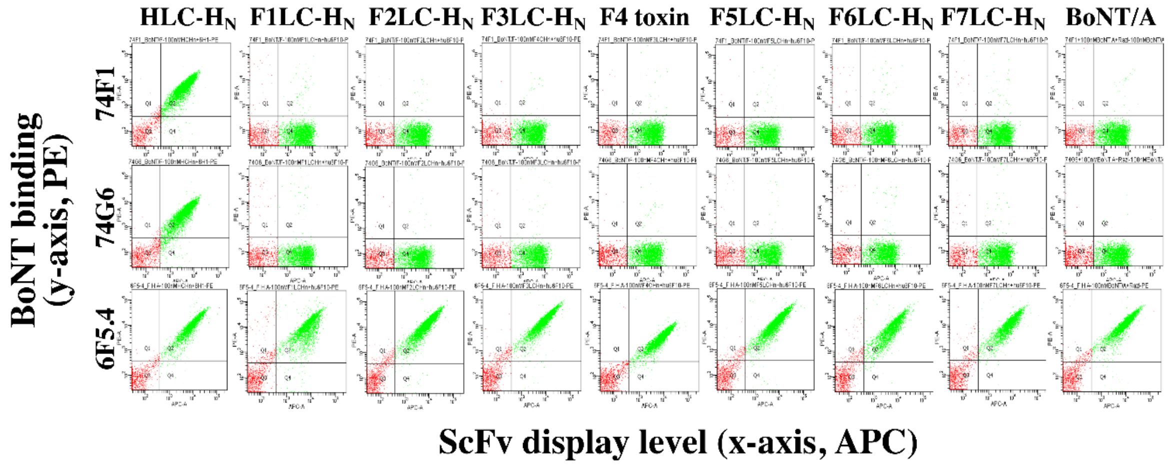

Figure S5. Dot plots of the binding of BoNT/H HN scFv to BoNT/F subtypes and BoNT/A. Yeast-displayed BoNT/H HN scFv 74F1, 74G6 and 6F5.4 were incubated with 100 nM of the indicated BoNT/F LC-HN subtype, BoNT/F4 holotoxin, BoNT/A holotoxin or BoNT/ HLC-HN. BoNT/F subtype binding was detected with IgG hu6F10, BoNT/H LC-HN binding was detected with IgG 6H1 and BoNT/A holotoxin binding was detected by IgG RAZ1.

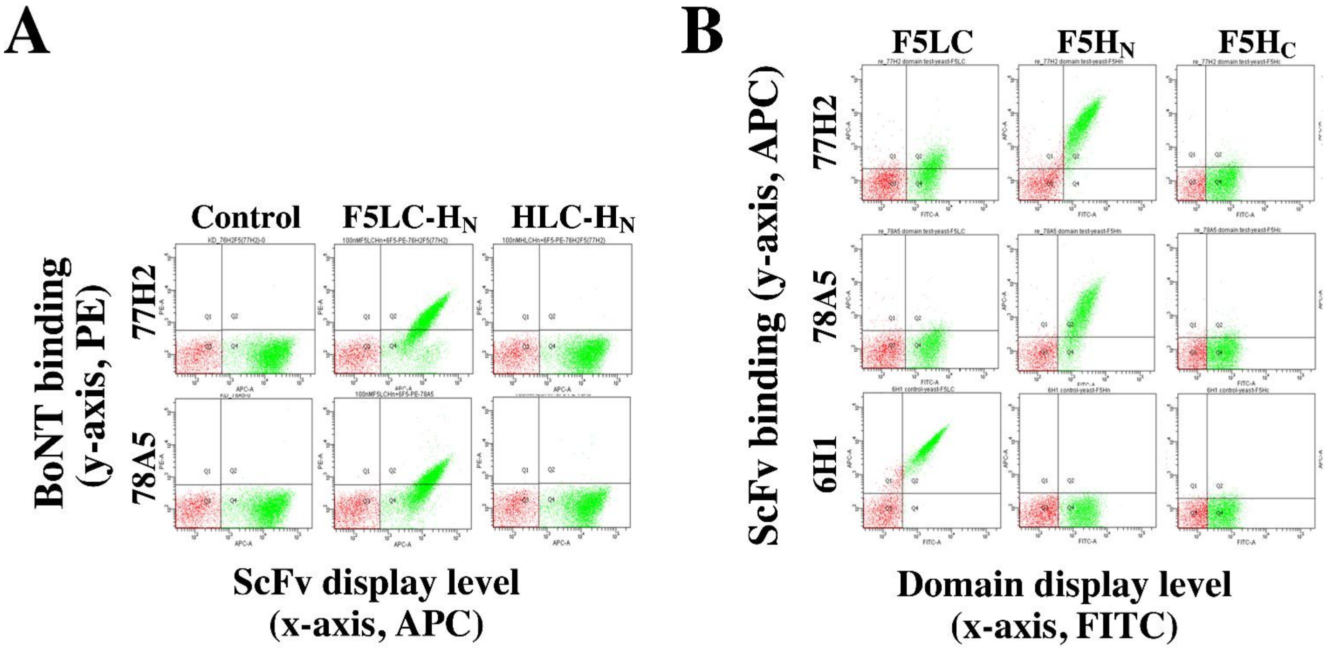

Figure S6. BoNT/F5 LC-H_N scFv 77H2 and 78A5 bind the H_N domain and do not bind BoNT/H LC-H_N. (**A**) Yeast-displayed scFv 77H2 and 78A5 were incubated with 100 nM BoNT/F5 LC-H_N or BoNT/H LC-H_N and binding was detected by using flow cytometry and mAb 6F5.4. (**B)** Yeast-displayed BoNT/F5LC, H_N or H_C were incubated with 100 nM of soluble scFv 77H2, 78A5 followed by anti-myc tag IgG (9E10) and goat anti-mouse IgG1 for detection. AlexaFluor-647 conjugated IgG 6H1 was included as a control showing LC domain binding.

References

1. Fan, Y.; Barash, J.R.; Lou, J.; Conrad, F.; Marks, J.D.; Arnon, S.S. Immunological characterization and neutralizing ability of monoclonal antibodies directed against botulinum neurotoxin type H. *J. Infect. Dis.* **2016**, *213*, 1606–1614.
